# On the Transformation of Calomel into Corrosive Sublimate

**Published:** 1840-10

**Authors:** 


### *On the transformation of Calomel into Corrosive Sublimate.* By M. Mialhe.

I have the honor to communicate to the Society of Pharmacy, the summary of some experiments which I have made on the transformation of calomel into corrosive sublimate, experiments which I was suddenly forced to interrupt.

The point from which I started with my researches, was the following fact, reported by Vogel. A physician prescribed for a child twelve papers, each containing five grains of sal ammoniac, five grains of sugar, and half a grain of calomel: the child having died after taking several of the powders, the apothecary was accused of having committed an error in compounding the prescription. Luckily for our colleague, the accusation which hung over him was of short duration, Peter Koffer having quickly proven that, in presence of sal ammoniac and of water, calomel is partially changed into corrosive sublimate. This fact, of which I have ascertained the exactness, has always appeared to me very remarkable, and well worthy of fixing the attention of physicians and physiologists. It would not be so, if the assertion of one of the most distinguished professors of our school were founded in fact. This professor asserts to have proven, by means of experiment, that the chemical change of protochloride of mercury into deutochloride, does not take place under the circumstances stated by the German chemist. I shall not attempt to point out whence is the source of error into which our learned colleague has fallen; I shall at present content myself with publishing the conclusions which result from my experiments.

1. The protochloride of mercury, in presence of hydrochlorate of ammonia, or of the chlorides of sodium or potassium, and of pure distilled water, is changed partly into deutochloride of mercury, and into metallic mercury. This change takes place at the temperature of the human body, and even at common temperatures, and demands but few moments of contact to be effective. It is sufficient, for example, to be convinced of this fact, to allow calomel to remain a few minutes in the mouth; a mercurial taste, of sufficient intensity, will not be slow in exhibiting itself. This taste is the result of the mutual reaction of the chloride of mercury, and the alkaline chlorides in the saliva.

2. It is to the change of calomel into corrosive sublimate, and metallic mercury under the influence of sea-salt and the salts of ammonia, which we know to exist in the liquids of the alimentary canal, that we must attribute the pathological phenomena of mercurial salivation, from the administration of calomel. What proves that this is really the case, is, that when the protochloride of mercury does not purge, but is retained for a long time in the digestive tube, it excites an unusual secretion from the salivary glands, and this on account of the large quantity of corrosive sublimate which is produced.

The same phenomena happens after the long continued use of the protochloride of mercury, and from the same cause.

3. As the quantity of corrosive sublimate formed can only be proportional to the amount of alkaline chlorides which are contained in the viscera, those persons who eat large quantities of common salt, every thing else being equal, should be more susceptible than others, when under a mercurial course of medicine.

4. The antisyphilitic properties are communicated to it, either in whole or in part, by the sublimate and the mercury to which its chemical decomposition gives rise. It is, without doubt, the same as regards its anthelmintic virtues; it is by producing poisonous effects on the ascarides, by means of the two agents mentioned, that the mercurial chloride relieves us from these importunate guests.

5. All that has been said of the medicinal action of calomel may likewise be predicated of the prot-iodide of mercury, which, under the same circumstances, is converted into deut-iodide.—*Journ. de Pharm.*—*American Journal of Pharmacy.*

